# Temporal swelling: beyond giant cell arteritis

**DOI:** 10.1093/rap/rkad090

**Published:** 2023-10-18

**Authors:** Joana Martins-Martinho, Ana T Melo, Ana R Lopes, Diana P Afonso, Cristina Ponte

**Affiliations:** Rheumatology Department, Centro Hospitalar Universitário Lisboa Norte, Centro Académico de Medicina de Lisboa (CAML), Lisbon, Portugal; Instituto de Medicina Molecular, Faculdade de Medicina, Universidade de Lisboa, CAML, Lisbon, Portugal; Rheumatology Department, Centro Hospitalar Universitário Lisboa Norte, Centro Académico de Medicina de Lisboa (CAML), Lisbon, Portugal; Instituto de Medicina Molecular, Faculdade de Medicina, Universidade de Lisboa, CAML, Lisbon, Portugal; Rheumatology Department, Centro Hospitalar Universitário Lisboa Norte, Centro Académico de Medicina de Lisboa (CAML), Lisbon, Portugal; Instituto de Medicina Molecular, Faculdade de Medicina, Universidade de Lisboa, CAML, Lisbon, Portugal; Radiology Department, Hospital da Luz, Lisboa, Portugal; Radiology Department, Hospital Particular da Madeira, Funchal, Portugal; Rheumatology Department, Centro Hospitalar Universitário Lisboa Norte, Centro Académico de Medicina de Lisboa (CAML), Lisbon, Portugal; Instituto de Medicina Molecular, Faculdade de Medicina, Universidade de Lisboa, CAML, Lisbon, Portugal

A 60-year-old woman, with a background of HIV type 1 infection suppressed by anti-retroviral treatment, presented to the rheumatology clinic with a localized left temporal headache spanning 6 months. She denied visual anomalies, jaw claudication, weight loss and symptoms of PMR or suggestive of infection. On physical examination, she had a swelling in the temporal artery region (Fig. 1A), which was tender to the touch. Temporal artery pulses were present and symmetrical. The inflammatory markers were within the normal range. GCA was suspected, and an immediate ultrasound examination of the temporal and axillary arteries was performed, showing no signs compatible with this diagnosis. However, it revealed the presence of three well-defined oval-shaped hypoechoic images with fatty hilum in the left temporal region, suggestive of lymph nodes (Fig. 1B–D). Cranial CT confirmed the presence of unspecific infra-centimetric nodules in the left frontal epicranial soft tissue. The patient refused a biopsy. In conclusion, this case portrays a typical manifestation of GCA that was proved to be lymphadenopathy of unknown cause, in an unusual location. Ultrasound is useful in rapidly assessing a suspected GCA diagnosis [1], thus avoiding unnecessary glucocorticoid treatment and the delay of further appropriate investigations.

**Figure 1.**
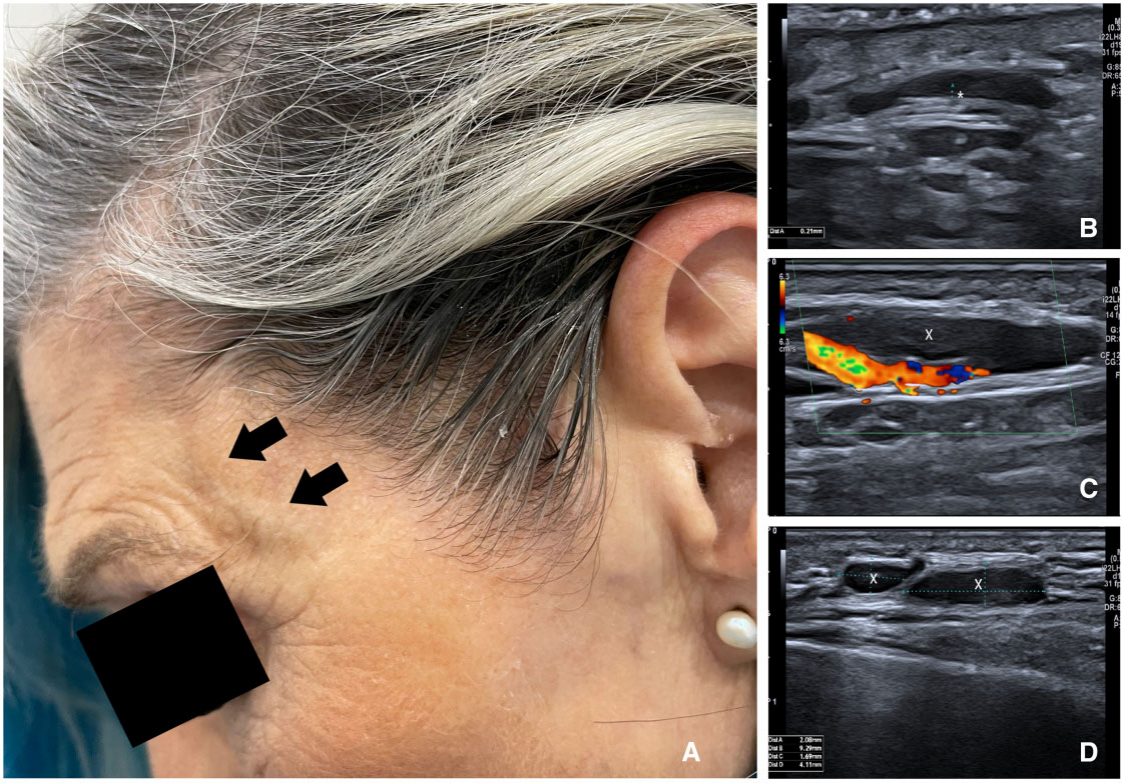
Patient with temporal swelling and its ultrasound evaluation. (**A**) Swelling in the left temporal area (arrows). (**B**) Ultrasound image of the left common superficial temporal artery, in transverse view, showing a normal intima–media complex with no halo sign (*). (**C**) Ultrasound image of a normal frontal branch of the left temporal artery, in transverse view, surrounded by a hypoechoic ovoid node (x). (**D**) Ultrasound image of two hypoechoic nodes with fatty hilum suggestive of lymph nodes

## Data Availability

The data underlying this article will be shared on reasonable request to the corresponding author.
